# Combination of time-restricted feeding with resistance exercise ameliorates MAFLD and improves glycemic homeostasis in obese mice

**DOI:** 10.1093/lifemeta/loaf023

**Published:** 2025-06-10

**Authors:** Guilherme Domingos Brisque, Ana Paula Azevêdo Macêdo, Guilherme Soderini Erlich, Gustavo Almeida Iasniswski, Vítor Rosetto Muñoz, Diego Brunelli, Alisson Luiz da Rocha, Guilherme Correia Ferri Antonio, Larissa Moreira Dias, Eduardo Rochete Ropelle, Dennys Esper Cintra, Adelino S R da Silva, José Rodrigo Pauli

**Affiliations:** Laboratory of Molecular Biology of Exercise (LaBMEx), University of Campinas (UNICAMP), Limeira, São Paulo 13484-350, Brazil; Laboratory of Molecular Biology of Exercise (LaBMEx), University of Campinas (UNICAMP), Limeira, São Paulo 13484-350, Brazil; Laboratory of Molecular Biology of Exercise (LaBMEx), University of Campinas (UNICAMP), Limeira, São Paulo 13484-350, Brazil; Laboratory of Molecular Biology of Exercise (LaBMEx), University of Campinas (UNICAMP), Limeira, São Paulo 13484-350, Brazil; Postgraduate Program in Rehabilitation and Functional Performance, Ribeirão Preto Medical School, University of São Paulo (USP), Ribeirão Preto, São Paulo 14040-900, Brazil; Postgraduate Program in Physical Education and Sport, School of Physical Education and Sport of Ribeirão Preto, University of São Paulo (USP), Ribeirão Preto, São Paulo 14040-900, Brazil; Laboratory of Molecular Biology of Exercise (LaBMEx), University of Campinas (UNICAMP), Limeira, São Paulo 13484-350, Brazil; Laboratory of Molecular Biology of Exercise (LaBMEx), University of Campinas (UNICAMP), Limeira, São Paulo 13484-350, Brazil; Laboratory of Molecular Biology of Exercise (LaBMEx), University of Campinas (UNICAMP), Limeira, São Paulo 13484-350, Brazil; Laboratory of Molecular Biology of Exercise (LaBMEx), University of Campinas (UNICAMP), Limeira, São Paulo 13484-350, Brazil; Laboratory of Molecular Biology of Exercise (LaBMEx), University of Campinas (UNICAMP), Limeira, São Paulo 13484-350, Brazil; Laboratory of Cell Signaling, Obesity and Comorbidities Research Center (OCRC), University of Campinas, Campinas, São Paulo 13083-864, Brazil; National Institute of Science and Technology of Obesity and Diabetes, University of Campinas (UNICAMP), Campinas, São Paulo 13083-887, Brazil; Laboratory of Cell Signaling, Obesity and Comorbidities Research Center (OCRC), University of Campinas, Campinas, São Paulo 13083-864, Brazil; Laboratory of Nutritional Genomics (LabGeN), University of Campinas (UNICAMP), Limeira, São Paulo 13484-350, Brazil; Laboratory of Nutritional Genomics (LabGeN), University of Campinas (UNICAMP), Limeira, São Paulo 13484-350, Brazil; Laboratory of Molecular Biology of Exercise (LaBMEx), University of Campinas (UNICAMP), Limeira, São Paulo 13484-350, Brazil; Laboratory of Cell Signaling, Obesity and Comorbidities Research Center (OCRC), University of Campinas, Campinas, São Paulo 13083-864, Brazil; National Institute of Science and Technology of Obesity and Diabetes, University of Campinas (UNICAMP), Campinas, São Paulo 13083-887, Brazil

**Dear Editor,**

Obesity is a pandemic, chronic, multifactorial disease that contributes to numerous conditions, including metabolic dysfunction-associated fatty liver disease (MAFLD) [1]. MAFLD is characterized by excessive triglyceride accumulation in the liver in the presence of at least one cardiometabolic risk factor (e.g. obesity, elevated blood pressure, insulin resistance, dyslipidemia, or elevated plasma glucose) and the absence of other apparent causes. Obese individuals may progress to the more severe condition of metabolic dysfunction-associated steatohepatitis (MASH), an advanced stage of MAFLD characterized by hepatic fat accumulation and inflammation. MAFLD represents the initial phase of liver disease, with MASH as the intermediate stage and fibrosis or cirrhosis as the final stage if left untreated [1].

Currently, MAFLD is the most prevalent liver disease, affecting approximately 30% of the global population [2]. Its prevalence is lower in premenopausal women compared to men, indicating sexual dimorphism [2]. Addressing MAFLD in the context of obesity remains a significant challenge due to gaps in understanding, particularly regarding sex-specific mechanisms. Traditional strategies, such as calorie restriction and increased physical activity, often fail to achieve sustainable long-term success [3]. Thus, novel interventions involving those well-known non-drug therapies to mitigate obesity and combat MAFLD are urgently needed.

Time-restricted feeding (TRF), which confines food intake to a limited daily window (6–12 h) followed by fasting, has garnered attention for its benefits in weight loss and metabolism in preclinical [4] and clinical studies [5]. These benefits are attributed to the alignment of eating patterns with circadian metabolic rhythms, countering the detrimental effects of unrestricted 24-h food consumption [6]. Notably, TRF often results in unintentional calorie reduction within the eating window [7]. Consequently, TRF offers a simple, practical, and cost-effective approach to obesity management without requiring substantial dietary changes. The findings of these studies have boosted research efforts aimed at translating the outcomes of TRF to clinical populations. Despite the growing number of studies investigating the effects of TRF in various clinical contexts, significant gaps remain. Notably, further research is needed to explore the effects of TRF in females and to assess its combination with other non-pharmacological interventions, such as physical exercise.

While physical exercise is widely recognized for its role in reducing body weight and liver fat, there is no consensus on the most effective type of exercise for steatosis treatment [8]. Resistance exercise may be advantageous for individuals with low physical capacity, a common condition in obesity, and is often better tolerated [9]. Combining TRF with resistance exercise could provide complementary benefits, yet few studies have explored the effects of this combination, particularly concerning hepatic lipogenesis signaling in both sexes. Thus, this investigation evaluates the impact of TRF and resistance exercise on obesity and MAFLD in male and female Swiss mice fed a high-fat diet (HFD).

Mice subjected to TRF had a 12-h feeding window (6:00 p.m.–6:00 a.m.), allowing unrestricted food access during this period. Animals were fed either a standard diet or an HFD *ad libitum* or with TRF, and HFD-fed mice underwent TRF, resistance training (RT), or a combination of both. RT involved progressive resistance exercise (weight-pulling) with sessions on alternate days (20 repetitions, 2-min rests between sets) at 70%−85% of the maximum voluntary carrying capacity (MVCC), conducted at 6:00 a.m. immediately after feeding. At 10 weeks of age, mice began the TRF and/or RT protocol, which lasted for 10 weeks. All analyses conducted in the study were performed 24 h after the last physical exercise session and, therefore, took place at ZT1 (07:00 a.m.). The summary of the experimental design is presented in Supplementary Fig. S1a and b. Physiological, histological, and molecular analyses revealed that TRF combined with RT provided complementary benefits compared to each intervention alone, with notable sex-specific differences. The results are shown in Fig. 1 and Supplementary Figs S1–S6.

**Figure 1:**
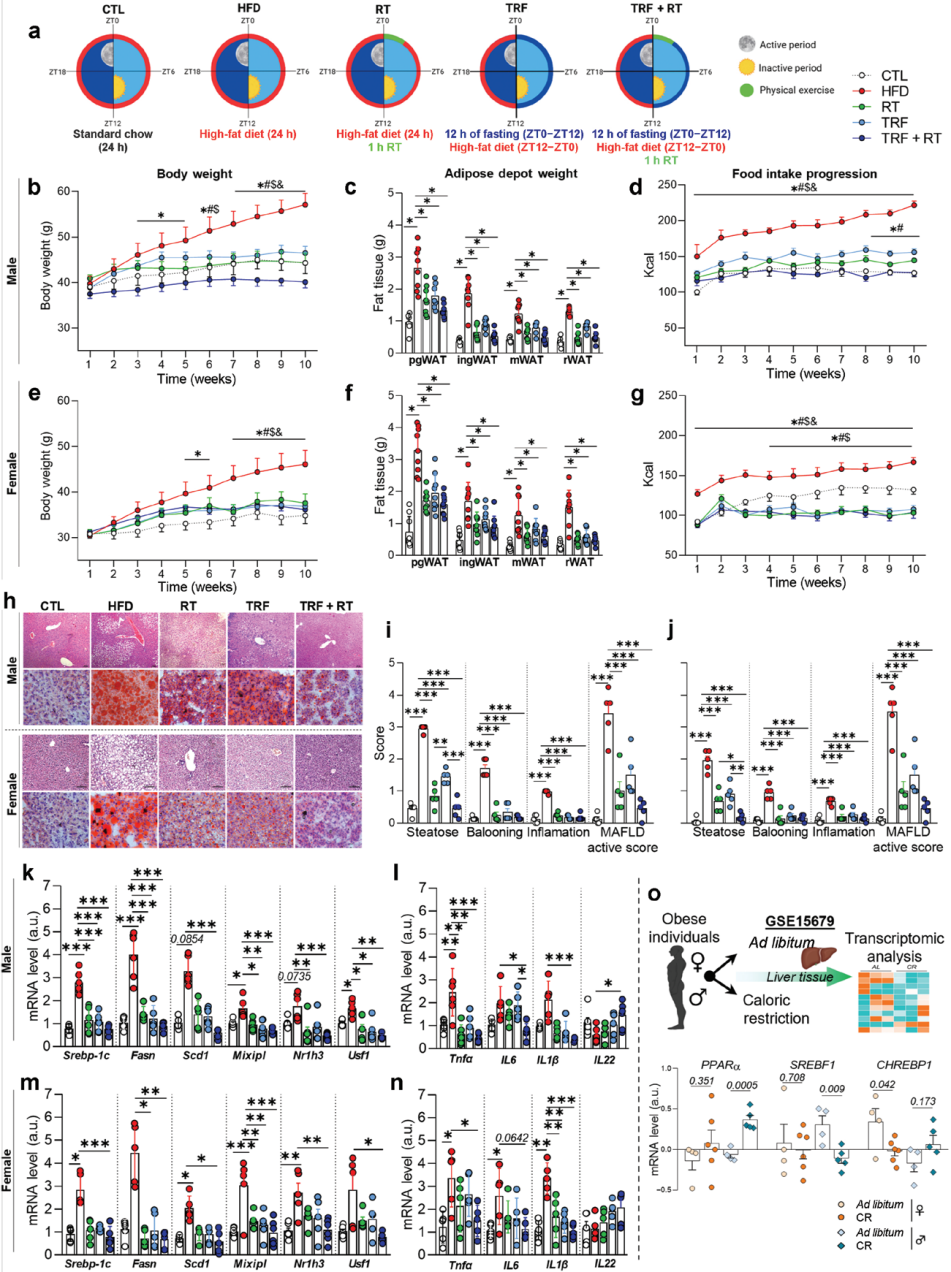
(a) Experimental design. (b–g) Body weight progression (b and e), visceral adipose tissue weight (c and f), and food intake (d and g). For male mice, *n* = 6–8; female mice, *n* = 9. (h) Histological liver tissue sections stained with hematoxylin–eosin (H&E) and Oil Red O. (i and j) MAFLD score in male (i) and female (j) mice (*n* = 5). (k and m) Lipogenic genes. (l and n) Inflammatory genes. (o) Liver transcriptomic data (*n* = 6). Bars represent the mean ± SEM. Statistical significance: *P < 0.05; **P < 0.01; ***P < 0.001. Comparisons: HFD versus CTL (#); HFD versus RT ($); HFD versus TRF (&); HFD versus TRF + RT (^*^); CTL versus RT, TRF, TRF + RT (*, #, $).

Skeletal muscle, accounting for approximately 40% of total body mass, plays a critical role in metabolism and energy expenditure. Male and female mice subjected to the RT protocol (RT and TRF + RT groups) demonstrated enhanced performance in various motor tests, including MVCC, rotarod, and hand grip force, compared to their sedentary counterparts [control (CTL), HFD, and TRF groups] (Supplementary Fig. S2a and f). Additionally, both sexes exhibited an increase in the cross-sectional area of the gastrocnemius muscle following RT, either alone or in combination with TRF. Interestingly, female mice subjected to TRF alone displayed a larger skeletal muscle cross-sectional area than females in the HFD group, a phenomenon not observed in male mice (Supplementary Fig. S2g and h). These findings suggest the presence of a sex-specific dimorphism in the effects of an HFD and TRF on skeletal muscle. A previous study from our laboratory showed that stair-climbing RT alone or combined with TRF preserves gastrocnemius muscle mass in male mice fed an HFD [9].

Male and female mice fed an HFD exhibited a significant increase in body weight compared to their control counterparts receiving a standard diet (Fig. 1b and e; Supplementary Fig. S3h and j). This finding aligns with previous studies demonstrating that an HFD induces weight gain and increases rodent adiposity [4, 9]. However, in both sexes, mice subjected to TRF or RT alone showed attenuated body weight gain compared to the HFD group. Notably, male mice exhibited a further reduction in body weight gain when TRF and RT were combined, a phenomenon not observed in female mice. TRF and RT also reduced white adipose tissue (WAT) depots (e.g. perigonadal, inguinal, mesenteric, and retroperitoneal WAT) in both sexes. Previous studies also support the ability of TRF to reduce body weight in mice [4, 9] and humans [5]. However, male mice in the TRF + RT group had a smaller adipocyte area than TRF or RT alone, a finding absent in females (Supplementary Fig. S3g and i). These results suggest a synergistic effect of TRF and RT in reducing fat mass and improving metabolic parameters, particularly in males. Female mice exhibit greater resistance to weight gain and increased adiposity in response to an HFD than male mice, which may partially explain the lower impact of the TRF + RT combination observed in female mice in our study. The present findings demonstrate that weight gain in female mice was lower than that in males. Additionally, images of the perigonadal adipose tissue (PgWAT) and measurements of mean adipocyte area reveal that the combined intervention (TRF + RT) was more effective in reducing adiposity than either intervention alone (TRF or RT) in male mice. However, this effect was not observed in female mice (Supplementary Fig. S3g and i).

Regarding carbohydrate metabolism, both sexes showed improvements with TRF (Supplementary Fig. S3a and b). Male mice in the RT and TRF + RT groups exhibited better glucose tolerance than TRF alone (Supplementary Fig. S3a). These findings emphasize the importance of aligning interventions with circadian rhythms to optimize glucose metabolism. In female mice, glucose tolerance was significantly improved in both the isolated (TRF or RT) and combined (TRF + RT) interventions compared to the HFD group (Supplementary Fig. S3d). Enhanced insulin sensitivity was observed across interventions in both sexes, with women generally displaying a natural advantage against insulin resistance, partly due to differences in fat distribution [2, 10]. In our study, male and female mice fed an HFD exhibited reduced insulin sensitivity compared to their control counterparts fed a standard diet (Supplementary Fig. S3b and e). Conversely, mice of both sexes subjected to TRF, RT, or the combined TRF + RT intervention showed improved insulin sensitivity relative to the HFD group.

Additionally, fasting blood glucose levels were elevated in HFD-fed mice. Still, both the isolated (TRF or RT) and combined (TRF + RT) interventions effectively mitigated these adverse effects in both sexes (Supplementary Fig. S3c and f). Hepatic analyses revealed significant fat deposition in HFD-fed mice, which was mitigated by TRF, RT, and their combination in both sexes. However, only male mice in the TRF + RT group exhibited lower hepatic cholesterol levels than those subjected to TRF or RT alone (Supplementary Fig. S4a). In contrast, the isolated interventions (TRF or RT) and the combined TRF + RT intervention reduced hepatic triacylglycerol content in male and female mice (Supplementary Fig. S4b and h). These findings align with previous studies demonstrating reduced hepatic fat and lipogenesis markers through TRF [4, 11] and resistance exercise [8, 9]. These blood biochemistry results were accompanied by a reduction in the expression of genes related to cholesterol metabolism [sterol regulatory element-binding protein 2 (*Srebp2*), 3-hydroxy-3-methylglutaryl-coenzyme A reductase (*Hmgcr*), and squalene epoxidase (*Sqle*)] in the liver (Supplementary Fig. S4d and j) of both male and female mice subjected to the TRF and TRF + RT protocols. Furthermore, interventions involving TRF alone or in combination with RT led to decreased levels of serum alanine aminotransferase (ALT) and aspartate aminotransferase (AST) (Supplementary Fig. S4e, f, k, and l) in mice of both sexes. These enzymes are markers of potential liver injury. In male mice, the TRF + RT group exhibited a more pronounced reduction in ALT and AST levels compared to the TRF and/or RT groups.

The consumption of hypercaloric diet and HFD is linked to mitochondrial dysfunction and impaired liver metabolism [9]. Our study demonstrated that interventions such as TRF and RT, individually or mainly in combination (TRF + RT), enhanced hepatic mitochondrial respiratory function. Improvements included increased fatty acid oxidation capacity and coupled ATP synthesis respiration, regardless of sex, compared to the HFD group (Supplementary Fig. S4c and i). Notably, in male mice, isolated TRF was less effective in preserving basal mitochondrial respiration than the TRF + RT combination, emphasizing the synergistic effects of these interventions (Supplementary Fig. S4i). Damasceno de Lima *et al.* demonstrated that mice fed HFD exhibit impaired mitochondrial respiratory function [9]. A study by Fuller *et al*. showed that oxygen flow and mitochondrial coupling may not differ significantly between sexes under certain conditions but are influenced by hormonal factors, the estrous cycle, and estrogen-related genotypes [12]. Future investigations should account for these variables when analyzing mitochondrial function in female mice.

Liver transcriptomic analyses revealed elevated expression levels of lipogenic genes [sterol regulatory element-binding protein 1c (*Srebp1c*), fatty acid synthase (*Fasn*), stearoyl-CoA desaturase-1 (*Scd1*), max-like protein X (MLX)-interacting protein-like (*Mixipl*), nuclear receptor subfamily 1 group H member 3 (*Nr1h3*), and upstream stimulatory factor 1 (*Usf1*)] in HFD-fed male mice, and of *Srebp1c*, *Scd1*, *Mixipl*, and *Nr1h3* in HFD-fed female mice (Fig. 1k and m). Additionally, increased protein levels of SREBP-1c and FAS were observed in the livers of HFD-fed mice of both sexes (Supplementary Fig. S5a–d). These findings indicate that excessive lipogenesis is closely associated with disturbances in lipid homeostasis, potentially leading to pathological outcomes such as dyslipidemia and fatty liver disease [13]. In contrast, TRF and RT interventions reduced the expression of lipogenic markers, particularly when applied in combination. Previous evidence suggests that TRF and RT can attenuate the expression of lipogenic enzymes, thereby improving hepatic lipid profiles [9].

Analysis of liver transcriptomic data from obese humans under caloric restriction (GSE15679) revealed increased expression levels of peroxisome proliferator-activated receptor alpha (*PPARα*) and reduced sterol regulatory element-binding transcription factor 1 (*SREBF1*) in males, with additional carbohydrate response element-binding protein 1 (*CHREBP1*) downregulation in females, supporting sex-specific regulation of fatty acid metabolism (Fig. 1o). Future research should investigate the effects of TRF combined with RT performed in the fasting state. In the present study, the RT protocol was conducted immediately after the feeding window, at the beginning of the light phase. Evaluating the impact of this intervention when implemented at the onset of the dark cycle, during the fasting period, may offer deeper insights into its physiological effects in both sexes.

Furthermore, a sex-specific regulation of inflammatory gene expression [tumor necrosis factor α (*Tnf-α*), interleukin-6 (*Il6*), *Il1-α*, and *Il22*] was observed (Fig. 1l and n) in the liver. The combined intervention (TRF + RT) was more effective in reducing *Tnf-α*, *Il1-β*, and *Il6* expression in both sexes. In male mice, the TRF-only group exhibited higher *Il6* expression compared to the TRF + RT group, suggesting a differential effect of this approach on the inflammatory response. These findings highlight the potential of TRF + RT as a therapeutic strategy to modulate inflammation. Regarding *Il22* expression, a member of the IL-10 cytokine family with hepatoprotective properties [14], an increase was observed only in the TRF + RT group of male mice, compared to the HFD, TRF, and RT groups. Further studies assessing *Il22* levels in response to TRF and RT interventions will be essential to clarify the role of this cytokine and its potential protective effects in metabolic dysfunction-associated steatotic liver disease (MASLD).

Both TRF and physical exercise exert pleiotropic and multi-organ effects, likely sharing some overlapping mechanisms. However, it is intriguing to hypothesize that each also employs tissue-specific and pathway-specific mechanisms (e.g. exercise may more strongly stimulate adipose tissue lipolysis or increase energy expenditure). These distinct mechanisms could confer additive benefits, potentially leading to a more robust therapeutic response when combined.

In conclusion, combining TRF with RT synergistically decreased central adiposity and hepatic lipids in mice of both sexes, with significant reductions in lipogenesis, cholesterol, and inflammatory gene expression. These findings highlight the potential of TRF + RT as a strategy to prevent obesity and MAFLD, irrespective of sex.

## Limitations of the study

While the findings on the combination of TRF and exercise are compelling, our study has limitations, including its descriptive nature and lack of mechanistic experiments linking TRF + RT to observed metabolic improvements. RT’s systemic effects likely contribute to enhanced glucose uptake in skeletal muscle and improved insulin sensitivity (Supplementary Fig. S6a–d). Future studies should explore molecular mechanisms in skeletal muscle and adipose tissue.

## Supplementary Material

loaf023_suppl_Supplementary_Figures_S1-S6

## Data Availability

The authors confirm that all the data supporting the findings of this study are available within the supplementary material and corresponding authors.
