# Supplementary material for: Combination of time-restricted feeding with resistance exercise ameliorates MAFLD and improves glycemic homeostasis in obese mice
Source: Life Metab. 2025 Jun 10;4(4):loaf023. doi: 10.1093/lifemeta/loaf023 (PMC12230717; doi:10.1093/lifemeta/loaf023)
Supplement: loaf023_suppl_Supplementary_Figures_S1-S6 [file loaf023_suppl_supplementary_figures_s1-s6.docx]

Supplementary Materials for

**Combination of time-restricted feeding with resistance exercise ameliorates MAFLD and improves glycemic homeostasis in obese mice**

**Materials and methods**

**Animals and ethical procedures**

Male Swiss mice, aged 10 weeks and sourced from the Central Animal Facility at UNICAMP (CEMIBE), were used for this research. All procedures and experiments involving animals were conducted following Brazilian legislation on the scientific use of animals (Law No. 11,794, dated October 8, 2008). Before the start of experimental procedures, all protocols were submitted for review by the Ethics Committee on Animal Use (CEUA) of the Institute of Biological Sciences, UNICAMP – Campinas-SP. The mice were individually housed in polyethylene cages under controlled 12-h light/12-h dark cycle conditions, with free access to water and either a standard or high-fat diet (HFD). The standard diet was a commercial pellet provided by Nuvilab (Quimtia, Colombo, PR, Brazil), with a nutritional composition of 23% crude protein, 4% lipids, and 5% fiber. The HFD composition included 11.55% corn starch, 20% casein, 10% sucrose, 13.2% dextrinized starch, 4% soybean oil, 31.2% lard, 5% cellulose, 3.5% mineral mix, 1% vitamin mix, 0.3% L-cystine, and 0.25% choline bitartrate, based on the American Institute of Nutrition (AIN-93G) guidelines.

# Animals and experimental groups

The Swiss mice, both males and females, were distributed into five experimental groups: ⅰ) Control group (CTL): mice that received standard diet *ad libitum*; ⅱ) Diet-induced obese (OB) mice that were fed with an HFD *ad libitum*; ⅲ) Mice submitted to time-restricted feeding (TRF), fed with an HFD for 12 h followed by 12 h of food restriction; ⅳ) Mice submitted to the resistance training (weight pulling exercise training protocol) (RT), fed with an HFD *ad libitum*; ⅴ) Mice submitted to TRF combined with RT (TRF + RT), fed with an HFD for 12 h followed by 12 h of food restriction. The analyses described below were performed after a 4-h fasting and 24 h after the last exercise session**.** Fasting blood glucose was measured after a 12-h fasting period. The experimental design was laid out in Supplementary Fig. S5a.

**Human public dataset**

Liver transcriptomic data (GSE15679) from obese individuals subjected to severe caloric restriction were analyzed to evaluate the expression of *PPARα*, *SREBF1*, and *CHREBP1* genes. An unpaired *t*-test was performed to compare the effects of caloric restriction between male and female subjects.

# TRF protocol

The TRF protocol was conducted as described below. Zeitgeber time (ZT) 0 was defined as the start of the light (inactive) cycle, and ZT12 as the end of the light cycle and the beginning of the dark (active) cycle. Animals subjected to TRF had access to food from ZT12 until the end of ZT24, allowing for 12 h of unrestricted access to food (from 6:00 p.m. to 6:00 a.m.). After the food was removed at ZT24, the animals underwent a restricted feeding period until ZT12, resulting in 12 h of restricted feeding (no food access). TRF was implemented 5 days a week, while on Saturday and Sunday, the animals had free access to food. No water restriction was imposed on the animals in this study. An experimental design summarizing the TRF period can be seen in Fig. 1a.

# Apparatus used for the resistance training protocol

The resistance exercise protocol utilized a custom-built wooden track covered with non-slip material (non-woven polypropylene fabric). Mice performed weighted running exercises using a traction cart in a safe and controlled manner. The track measured 150 cm in length, 10 cm in width, and 10 cm in height, with the final 25 cm consisting of a dark-colored protective and resting area made from medium-density fiberboard (MDF). Adaptation to the apparatus was conducted over 5 days. During the first trial, mice were placed 15 cm from the entrance to the covered section. A gentle manual stimulus (a light tap on the gluteal region with a plastic spoon) was applied if needed. In subsequent trials, mice were positioned progressively farther from the covered section: 30 cm and 60 cm in the second and third trials, respectively, and 90 cm in the fourth and fifth trials. During the adaptation period, mice were allowed to rest for 60 s in the covered section between trials.

# Determination of the maximum voluntary carrying capacity (MVCC)

The test started 48 h after the adaptation period. The first set involved pulling an unweighted cart to acclimate the animals. Subsequently, three sets were performed with progressively heavier weights of 300 g, 600 g, and 750 g. After that, 50 g increments were added for each set until the mouse could no longer complete the 90-cm track, indicating exhaustion. After each attempt, the animal rested for 5 min in a covered area. The highest weight successfully pulled by the mouse was recorded as the MVCC. The test was carried out before the start and in the eighth week of the experiment.

# RT: weight pulling training

Tethered running training started 48 h after determining the MVCC. Training was conducted on alternate days for 10 weeks, totaling 35 sessions. Each session began with a warm-up involving cart pulling without additional weight. Following the warm-up, 20 runs were performed per session, starting with loads set at 70% of each animal’s MVCC during the first week and progressively increasing to 85% over 10 weeks. Each run covered a distance of 60 cm, with a 1-min rest interval between runs. Training sessions were held at 6:00 a.m. (ZT0), immediately following the feeding period, to ensure sufficient energy availability for physical exertion while promoting energy expenditure and skeletal muscle activity. The experimental design is illustrated in Supplementary Fig. S5b. It is important to note that all analyses in this study were carried out 24 h after the last physical exercise session (ZT1).

# Grip strength test

The Grip Strength System (AVS Projetos®, São Carlos, São Paulo, Brazil) was used. Three preliminary grip attempts were conducted with the forepaws and three with the hind paws, during which no values were recorded. Each attempt involved pulling the animal by the tail so that both paws (fore or hind) passed through all the grid wires and detached. Subsequently, six valid attempts were performed, with the three highest tension values recorded in Newtons (N). The force exerted by the animal was measured using a grip-force sensor, which recorded values in gram-force (gf). These values were converted to Newtons by multiplying by 0.9800. The highest force applied to the metal bar was recorded as the peak tension (N) and used as the performance parameter. The test was conducted at baseline and during the eighth week of the experiment.

# Rotarod test

The rotarod test, a sensitive method for assessing motor coordination and balance in rodents, was conducted using an automated Rotarod device (model EFF 411, Insight®). The apparatus consisted of an acrylic box with an 8-cm diameter cylinder positioned horizontally, approximately 20 cm above the ground, and driven by a motor. The box was divided into four compartments, each 10 cm wide, allowing simultaneous testing of up to four mice. During the test, each mouse was placed on the rotating cylinder, which started at a speed of 10 rotations per minute and gradually increased to a maximum of 35 rotations per minute. The time each mouse maintained balance was recorded, up to a maximum of 10 min, or until the mouse fell. A floor impact detection system in each compartment automatically stopped the timer when the mouse fell. Mice were then returned to their compartments, and the timer was reset. The average of three attempts per mouse was recorded. The test was performed at baseline and during the eighth week of the experiment.

# Measurement of blood glucose

The blood was obtained from the animals’ tails to assess fasting blood glucose using a glucometer (Accu-Chek Active/Roche, Switzerland). Before the test, the mice were fasted for 12 h.

# Intraperitoneal insulin tolerance test (ITT)

Mice received an intraperitoneal injection of recombinant human insulin (Humulin R, Eli Lilly, Indianapolis, IN, USA) at a concentration of 1.5 U/kg body mass. Blood samples were collected from the tail at 0, 5, 10, 15, 20, 25, and 30 min to determine blood glucose levels. Time 0 corresponded to the baseline glucose level before insulin administration. The constant glucose uptake rate (KITT) was calculated using the formula 0.639/biological half-life (*t*_1/2_), where *t*_1/2_ was derived from the slope of the linear decay phase in glucose concentration based on least squares analysis. Blood glucose levels were measured throughout the test using a One Touch glucometer (Johnson & Johnson®).

# Intraperitoneal glucose tolerance test (IPGTT)

Blood glucose concentrations were measured from tail blood samples at 0, 15, 30, 60, 90, and 120 min using a One Touch glucometer (Johnson & Johnson®). Following the baseline measurement at time 0, a 50% glucose solution (2 g/kg body weight) was administered via intraperitoneal injection. Moreover, the area of the curve (AOC) for the glucose tolerance test (GTT) was presented, following the guidelines outlined previously [1].

# High-resolution respirometry

Hepatic oxygen consumption was measured using the Oxygraph-2k system (Oroboros Instruments, Innsbruck, Austria). Approximately 50 mg of liver tissues was homogenized in

0.5 mL of ice-cold MIR05 buffer (110 mmol/L sucrose, 60 mmol/L K-lactobionate, 0.5 mmol/L EGTA, 3 mmol/L MgCl₂, 20 mmol/L taurine, 10 mmol/L KH₂PO₄, 20 mmol/L HEPES, pH 7.1, and 0.1% BSA) using a pestle homogenizer, at a concentration of 1 mg/10 μL. From the homogenate, 2 mg (20 μL) of tissues was added to the oxygen chamber containing 2 mL of MIR05 buffer. Measurements were performed at 37°C. Malate (0.1 mmol/L) and glutamate (10 mmol/L) were supplemented into the chambers to evaluate complex I-dependent mitochondrial respiration. The respiratory stimulation of the ATP production pathway was assessed by adding ADP (2.5 mmol/L) and combined respiration of complexes I and II was measured by adding succinate (10 mmol/L). After that, mitochondrial coupling was investigated by inhibiting ATP synthase with oligomycin (2.5 μmol/L)

# Animal euthanasia and tissue collection

Twenty-four hours after the final exercise session and following a 2-h fasting period, mice were anesthetized via intraperitoneal injection of ketamine hydrochloride (90 mg/kg; Ketalar, Parke-Davis, Ann Arbor, MI) and xylazine (10 mg/kg; Rompun, Bayer, Leverkusen). All animals underwent anesthesia at the same circadian time (ZT1—7:00 a.m.) before being sacrificed by decapitation after the 10-week experimental protocol. Adipose depots (perigonadal, inguinal, mesenteric, and retroperitoneal), gastrocnemius muscle, and liver samples were carefully dissected and weighed using an analytical scale (Gehaka BK300). Perigonadal adipose tissue (PgWAT) was preserved in 4% paraformaldehyde (PFA), while liver and gastrocnemius muscle samples were rapidly frozen in liquid nitrogen and stored at −80°C for subsequent analyses. Liver and muscle fragments were processed for Quantitative real-time PCR (RT-qPCR) analysis, biochemical assays, and histological evaluation.

# Blood biochemistry analysis

The biochemical markers, including total cholesterol, triglycerides, alanine aminotransferase (ALT), and aspartate aminotransferase (AST) were measured in liver tissue samples using commercial kits (Laborlab, São Paulo, SP, Brazil) after tissue collection.

# Western blotting analysis

Liver tissue was homogenized in an extraction buffer containing 1% Triton X-100, 100 mmol/L Tris (pH 7.4), 100 mmol/L sodium pyrophosphate, 100 mmol/L sodium fluoride, 10 mmol/L EDTA, 10 mmol/L sodium vanadate, 2 mmol/L phenylmethylsulfonyl fluoride, and 0.1 mg/mL aprotinin at 4°C using a Bead Ruptor 12 Homogenizer (OMNI International, Kennesaw, GA, USA) at the maximum speed for 60 s. The homogenates were centrifuged at 12,851 g for 15 min at 4°C using an Eppendorf 5804R centrifuge (Hamburg, Germany) to remove insoluble debris. The supernatant was collected for further analysis, and protein concentration was determined using the bicinchoninic acid assay.

Laemmli buffer with 100 mmol/L dithiothreitol was added to the supernatant, and the samples were heated for 5−10 min (Laemmli, 1970). A total of 40 μg protein was loaded onto a polyacrylamide gel for SDS-PAGE separation, followed by transfer to nitrocellulose membranes. Transfer efficiency was confirmed by Ponceau staining. Membranes were blocked with 5% dry milk at room temperature for 1 h and incubated overnight at 4°C with primary antibodies, including FAS (#3189S), SREBP1c (#14492S), α-tubulin (#2144S), GLUT-4 (#2213), AKT (#9272) from Cell Signaling Technology (Danvers, MA, USA), and FAS (#48357), p-IRS-1/2 (Tyr612) (#17195), p-AKT (Ser473) (#48357), IRS-1 (#559), GAPDH (#47724) from Santa Cruz Biotecnology (Dalla, TX, USA).

Following primary antibody incubation, membranes were exposed to the corresponding secondary antibodies for 1 h. Protein bands were visualized using enhanced chemiluminescence, and band intensities were quantified via optical densitometry using UN-SCAN-IT gel 6.1 software (Silk Scientific, Inc., Orem, UT, USA).

# Histological perigonadal fat (PG) analysis

PG was carefully dissected after animal euthanasia, weighed on an analytical balance to determine the relative weight to body weight, and fixed in 4% PFA in PBS (pH 7.4) for 24 h. Following fixation, tissues were dehydrated in a graded ethanol series (70%−100%), clarified in xylene, and embedded in paraffin. Paraffin blocks were sectioned into 5-µm slices using a microtome and mounted on adhesive-coated glass slides, which were dried in an oven at 37°C.

The sections were deparaffinized, rehydrated, stained with hematoxylin (5 min) and eosin (2 min), and then mounted with coverslips using synthetic resin for microscopy. Slides were examined under an optical microscope with a digital camera, capturing images from three representative fields per slide. The captured images were analyzed using ImageJ software to calculate the average adipocyte area (µm²).

# Histological liver analysis

Liver tissue was extracted and cryopreserved in pre-cooled isopentane at −80°C. Thin sections (10 μm) were prepared using a Leica cryostat (CM1850, Heidelberg, Germany) and mounted on adhesion-treated slides. Sections were stained with Oil Red O solution (Sigma- Aldrich, St. Louis, MO, USA) for 30 min and counterstained with hematoxylin for 1 min. Slides were then washed and sealed with a gelatin-glycerin solution. Images were captured using Leica Application Suite software.

A small fragment of liver tissue was fixed in a 4% PFA solution for 48 h. The sample was dehydrated and embedded in paraffin to obtain the slices (at 7 µm) using a microtome (Leica ®, RM2145). Then, the slides were hydrated again and stained with hematoxylin-eosin (H&E). The images were acquired in an optical microscope at 10× and 40× magnification (Leica Microsystems) for the MAFLD Activity Score. It was assessed in a double-blind manner to evaluate diet-induced liver damage. Steatosis was graded on a scale of 0 to 3 based on the percentage of affected tissue; hepatocellular ballooning was graded from 0 to 2 based on the number of ballooning hepatocytes; and lobular inflammation was graded from 0 to 3 based on the number of inflammatory foci. These parameters were combined to calculate the total MAFLD Activity Score. Steatogenic profiles were assessed at 400× magnification, and inflammation was characterized by the presence of at least five inflammatory cells, not aligned in a row, across ten fields.

# Histological gastrocnemius muscle analysis

Following euthanasia, target muscles, including the gastrocnemius, were dissected and fixed in 4% PFA for 24 h. Tissues were dehydrated in a graded ethanol series (70%−100%), clarified in xylene, and embedded in paraffin. Thin sections (5 μm) were cut using a microtome and mounted on adhesive-coated slides.

Slides were deparaffinized, rehydrated, and stained with H&E for histological analysis. Images of the muscle sections were captured at 200× magnification using an optical microscope. The cross-sectional area of muscle fibers was measured using ImageJ software. Muscle hypertrophy was assessed by comparing the average fiber area between experimental and control groups, with an increase in cross-sectional area indicating hypertrophy.

# RNA extraction and RT-qPCR

Liver tissue and gastrocnemius muscle samples were homogenized in 400 μL TRIzol reagent (Thermo Fisher Scientific), and RNA was extracted following the manufacturer’s protocol. A total of 2 μg RNA was used for cDNA synthesis with the High-Capacity cDNA Reverse Transcription Kit (Thermo Fisher Scientific).

RT-qPCR analysis was performed using 300 ng of cDNA and 0.3 μmol/L primers targeting *Fasn*, *Srebp-1c*, *Scd1*, *Mixipl*, *Nr1h3*, *Usf1*, *Hmgcr*, *Sqle*, *Srebf2*, *Tnfα*, *Il6*, *Il1β*, and *Il22* for the liver, and *Glut-*4, *Akt*, and *Tbc1D4* for the gastrocnemius muscle, with *Gapdh* serving as the internal control (Supplementary Table S1). Primers were synthesized by Exxtend (Paulínia, SP, Brazil). Reactions were conducted using iTaq Universal SYBR Green Supermix (Bio-Rad, Hercules, CA, USA). Data analysis was performed using StepOne Software (Thermo Fisher Scientific) and expressed as ΔΔCt values.

| **Supplementary Table S1** Primer sequences used for RT-qPCR analysis. | | |
| --- | --- | --- |
| Gene | Forward primer | Reverse primer |
| **Liver**  *Fasn* | 5’-GAGGACACTCAAGTGGCTGA-3’ | 5’-GTGAGGTTGCTGTCGTCTGT-3’ |
| *Srebp1c* | 5’-GAGGACACTCAAGTGGCTGA-3’ | 5’-GGGAAGTCACTGTCTTGGTTGTT-3’ |
| *SCD1* | 5’-GGCGTGGGCTTCCAGAGGAG-3’ | 5’-GCCGAGCGGTGGCTTGTAAG-3’ |
| *Mixipl*  *Nr1h3*  *Usf1*  *Hmgcr*  *Sqle*  *Srebf2*  *Tnfα*  *Il1β*  *Il6*  *Il22* | 5’-ОССТССОССАСАССТСАСТС-3’  5’-ATCCCCTOCTGAAGACCTCTG-3’  5’-COTCTTCCGAACTGAGAATGGG-3’  5’-CTTGTGGAATGCCTTGTGATTG-3’  5’-ТСААССССАСТССАСТТСТС-3’  5’-GCGTTCTGGAGACCATGGA-3’  5’-GGTGCCTATGTCTCAGCCTCTT-3’  5’-TGGACCTTCCAGGATGAGGACA-3’  5’-ACCACTTCACAAGTCGGAGGC-3’  5’-GCTTGAGGTGTCCAACTTCCAG-3’ | 5’-AGTGCTGAGTTGGCGAAGGG-3’  5’-CTGCTTGGCAAAGTCTTCCCG-3’  5’-CTGGGTCATAGACTGAGTGGCA-3’  5’-AGCCGAAGCAGCACATGAT-3’  5’-GACTCCTTCAGGTGCTCAGG-3’  5’-ACAAAGTTOCTCTGAAAACAAATCA-3’  5’- GCCATAGAACTGATGAGAGGGAG-3’  5’-GTTCATCTCGGAGCCTGTAGTG-3’  5’-CTGCAAGTGCATCATCGTTGTTC-3’  5’-ACTCCTCGGAACAGTTTCTCCC-3’ |
| **Skeletal muscle**  *Glut-4* | 5’-ATCATCCGGAACCTGGAGG-3’ | 5’-CECTCACCOCOTTTAGACTC-3’ |
| *AKT* | 5’-ACCACCTGACCAAGATGACG-3’ | 5’-CCATCCCTCCAAGCTATCGT-3’ |
| *Tbc1D4* | 5’-CCACAACAGTCACGACCTCA-3’ | 5’-ACTGATCACATCTGGAACCTGG-3’ |
| *Gapdh* | 5’-AACTTTGGCATTGTGGAAGG-3’ | 5’-ACACATTGGGGGTAGGAACA-3’ |

# Statistical analysis

Data are presented as mean ± standard error of the mean (SEM). Normality of data distribution was assessed using the Shapiro-Wilk test. For normally distributed variables, one-way ANOVA was applied for comparisons between groups, and two-way ANOVA was used for analyses involving multiple factors or time points, followed by Tukey’s or Bonferroni *post-hoc* tests for multiple comparisons adjustment, as appropriate. Comparisons between two groups were performed using the Student’s *t*-test for independent samples. For non-normally distributed data, equivalent non-parametric tests were applied: Kruskal-Wallis for multiple groups and Mann-Whitney test for two-group comparisons. Statistical significance was set at *P* < 0.05. All statistical analyses and graphical representations were conducted using GraphPad Prism 8.0® software.

**Reference**

1. Virtue S, Vidal-Puig A. GTTs and ITTs in mice: simple tests, com­plex answers. *Nat Metab* 2021;**3**:883–6.

# Supplementary Figures

**
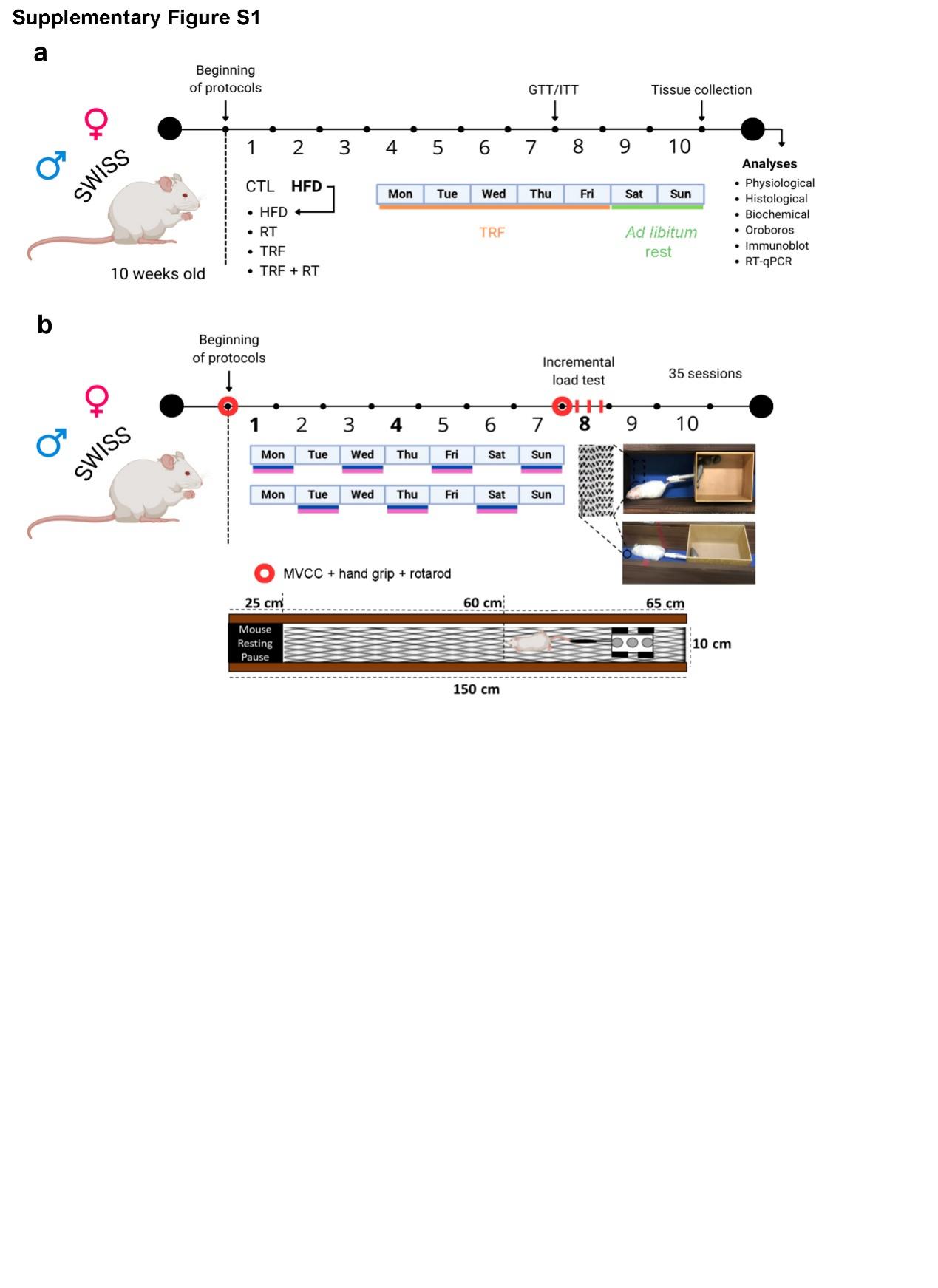
**

**Supplementary Figure S1** (a) The animals were distributed into five experimental groups: Control group (CTL): mice that received standard diet *ad libitum*; 2) Diet-induced obese (HFD) mice were fed with an HFD *ad libitum*; 3) Mice submitted to time-restricted feeding (TRF), fed with an HFD for 12 h followed by 12 h of food restriction; 4) Mice submitted to the resistance training (weight pulling) (RT), fed with an HFD *ad libitum*; 5) Mice submitted to TRF combined with RT (TRF + RT), fed with an HFD for 12 h followed by 12 h of food restriction. (b) Experimental design of the resistance exercise protocol (weight pulling) and performance assessments carried out during the experiment. The experimental period lasted for 10 weeks. GTT: glucose tolerance test; ITT: insulin tolerance test. MVCC: maximum voluntary carrying capacity.


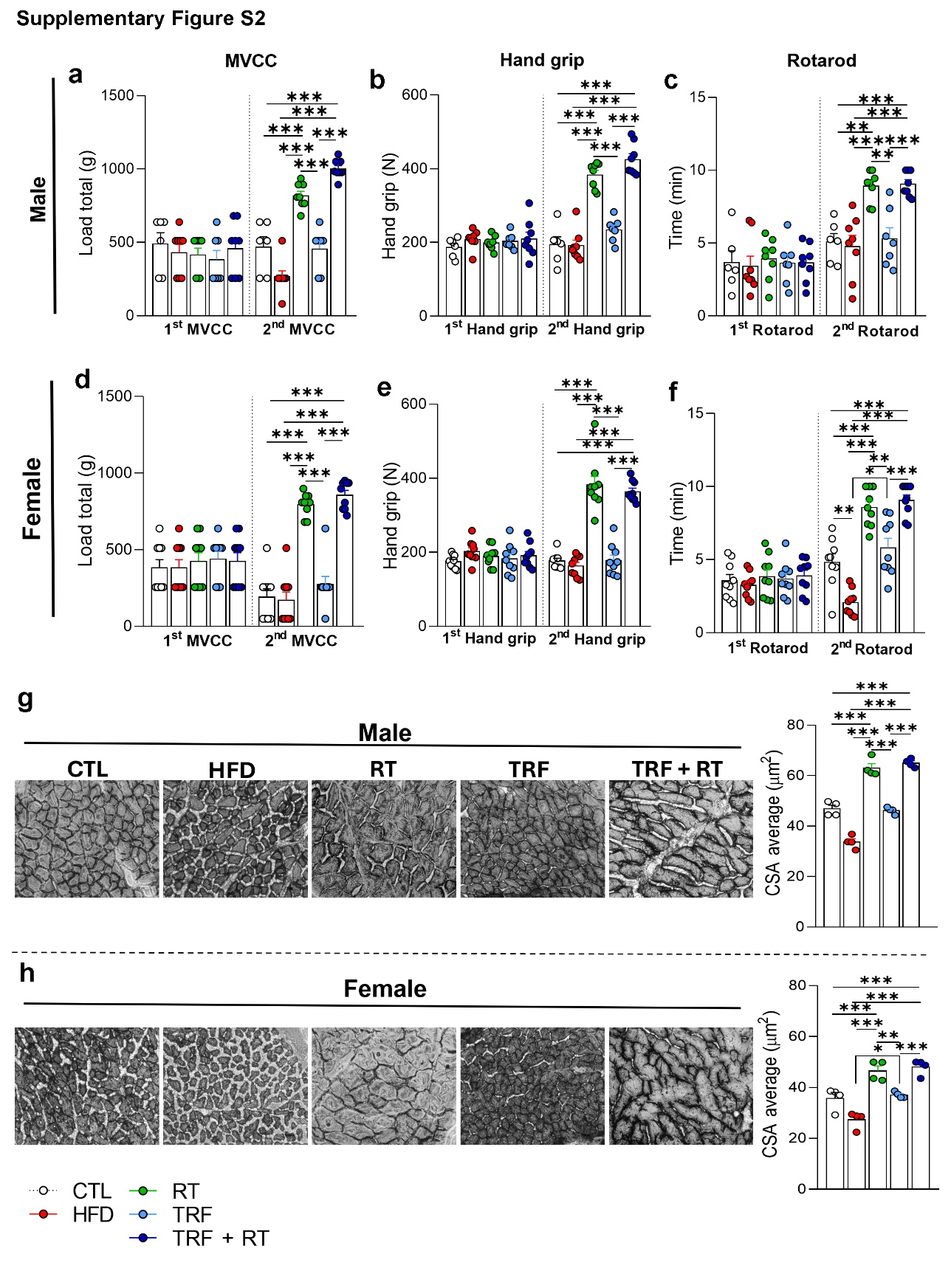


**Supplementary Figure S2** (a and d) Maximum voluntary contraction capacity (MVCC). (b and e) Hand grip test. (c and f) Rotarod test. (g and h) Representative H&E-stained images of the gastrocnemius muscle and average cross-sectional area (CSA) of muscle fibers (*n* = 4). Bars represent the mean ± SEM for the 1st and 2nd tests for male mice (*n* = 6−8) and female mice (*n* = 9) across different experimental groups: CTL (control), HFD (high-fat diet), RT (resistance training), TRF (time-restricted feeding), and TRF + RT (time-restricted feeding combined with resistance training). Statistical significance: ^*^*P* < 0.05; ^**^*P* < 0.01; ^***^*P* < 0.001.


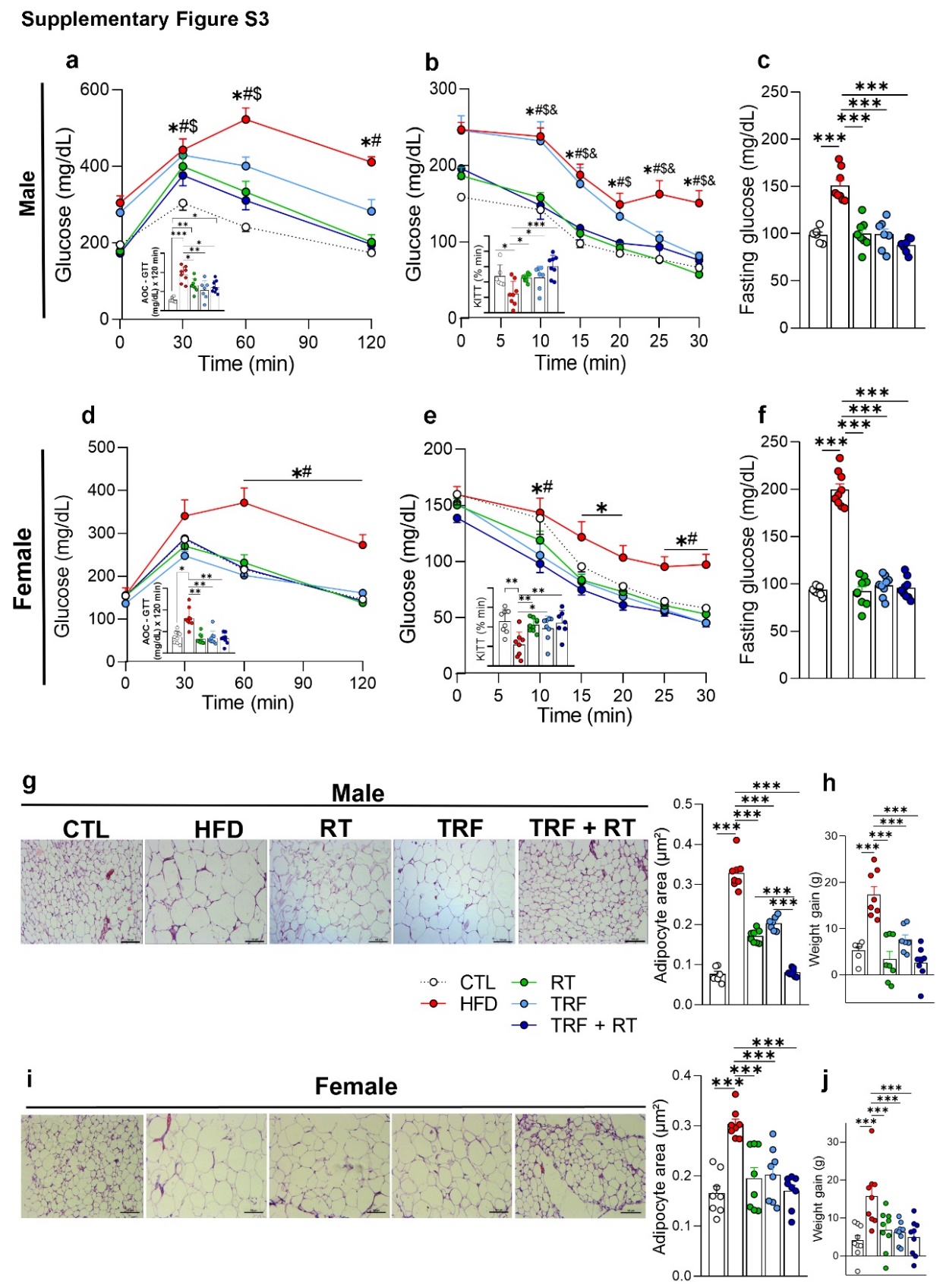


**Supplementary Figure S3** (a and d) Glucose tolerance test (GTT) and the area of the curve (AOC) for GTT. (b and e) Insulin tolerance test (ITT) and glucose decay rate (KITT). (c and f) Fasting blood glucose. (g and i) Representative H&E-stained images of the perigonadal adipose tissue (PgWAT) and mean adipocyte area. (h and j) Weight gain of the mice. Bars represent the mean ± SEM for male mice (*n* = 6−8) and female mice (*n* = 9) across different experimental groups: CTL (control), HFD (high-fat diet), RT (resistance training), TRF (time-restricted feeding), and TRF + RT (time-restricted feeding combined with resistance training). Statistical significance: ^*^*P* < 0.05; ^**^*P* < 0.01; ^***^*P* < 0.001. Males: CTL versus HFD, RT, and TRF (^✱^**^; #^**^;^ **^$^);** CTL versus HFD, and TRF (^✱^**);** HFD versus RT, TRF, and TRF + RT (**^#^**); TRF versus TRF + RT (**^$^**). Females: CTL versus HFD (^✱^); HFD versus RT, TRF, and TRF + RT (^#^).


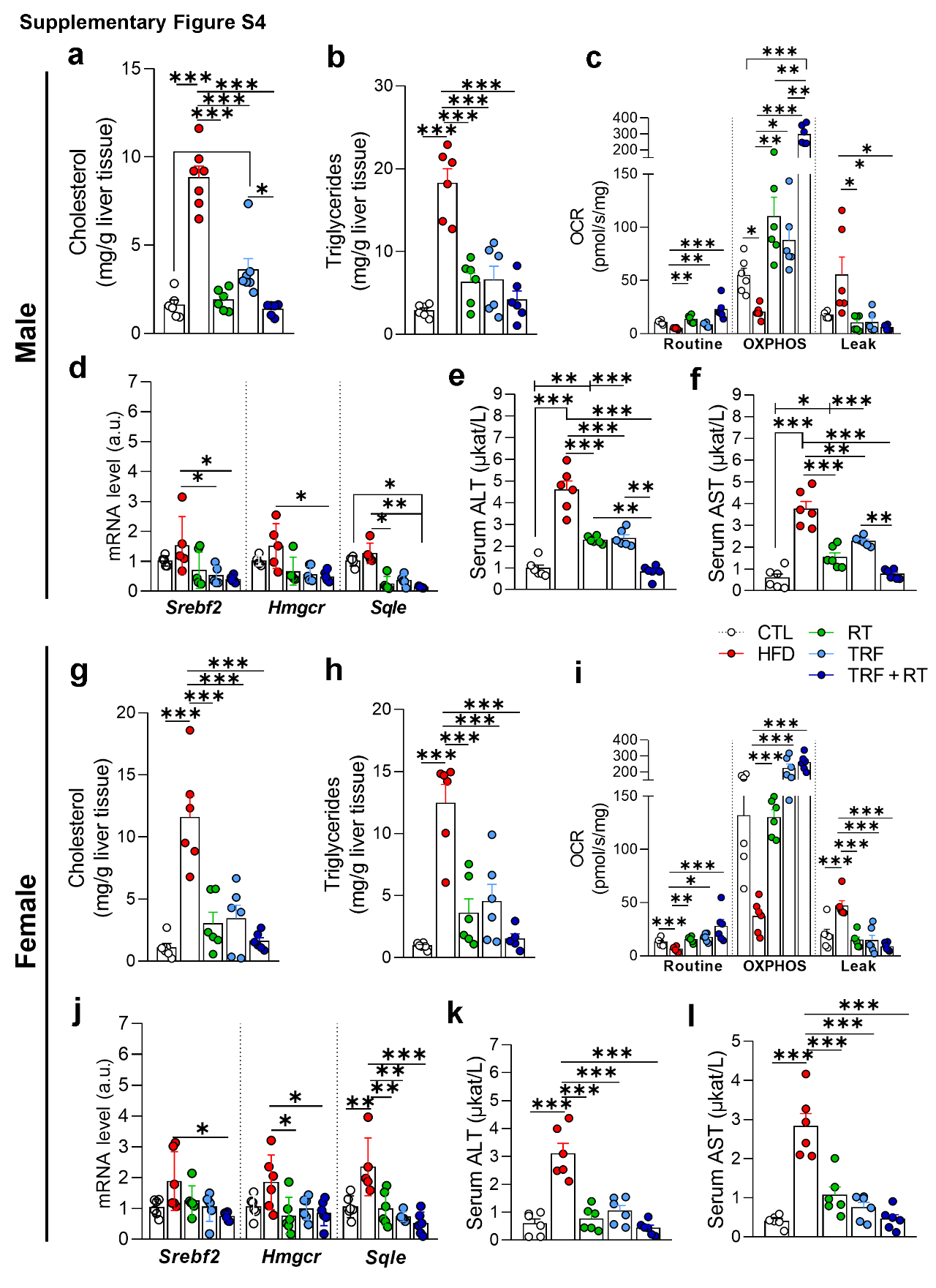


**Supplementary Figure S4** (a and g) Hepatic cholesterol in male and female mice. (b and h) Hepatic triglycerides in male and female mice. (c and i) Basal mitochondrial respiration, lipid oxidation capacity, and ATP-synthase-coupled respiration in the livers of male and female mice. (d and j) Expression of genes related to cholesterol metabolism in the livers of male and female mice. (e and k) Alanine aminotransferase (ALT) in male and female mice. (f and l) Aspartate aminotransferase (AST) in male and female mice. Bars represent the mean ± SEM for male and female mice (*n* = 6) across different experimental groups: CTL (control), HFD (high-fat diet), RT (resistance training), TRF (time-restricted feeding), and TRF + RT (time-restricted feeding combined with resistance training). Statistical significance: ^*^*P* < 0.05; ^**^*P* < 0.01; ^***^*P* < 0.001.


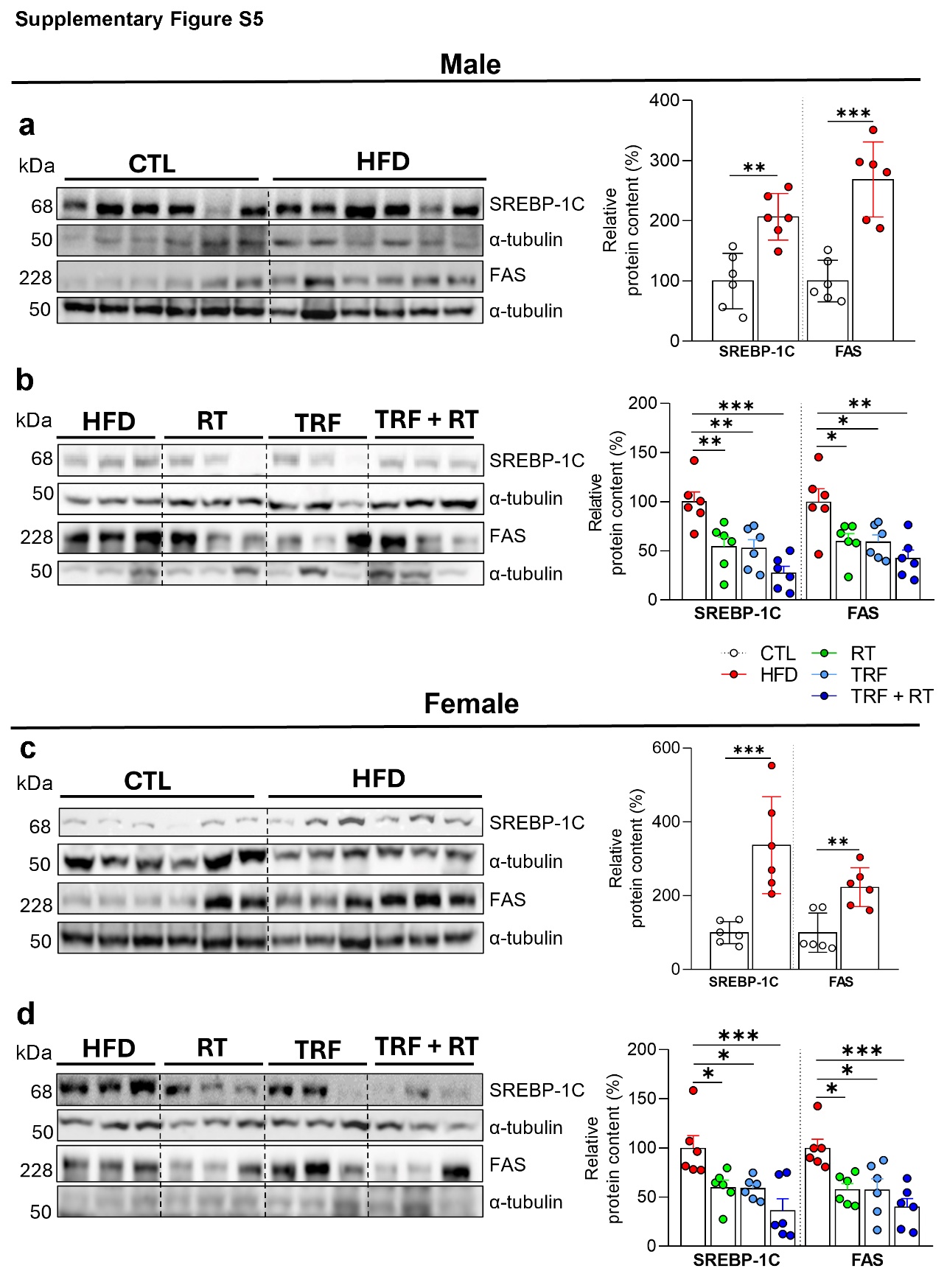


**Supplementary Figure S5** (a−d) Relative protein content in the livers of male and female mice. Bars represent the means ± SEM for male and female mice (*n* = 6) across different experimental groups: CTL (control), HFD (high-fat diet), RT (resistance training), TRF (time-restricted feeding), and TRF + RT (time-restricted feeding combined with resistance training). The α-tubulin protein was used as an assay control for SREBP-1C and FAS. Statistical significance: **P* < 0.05; ***P* < 0.01; ****P* < 0.001.


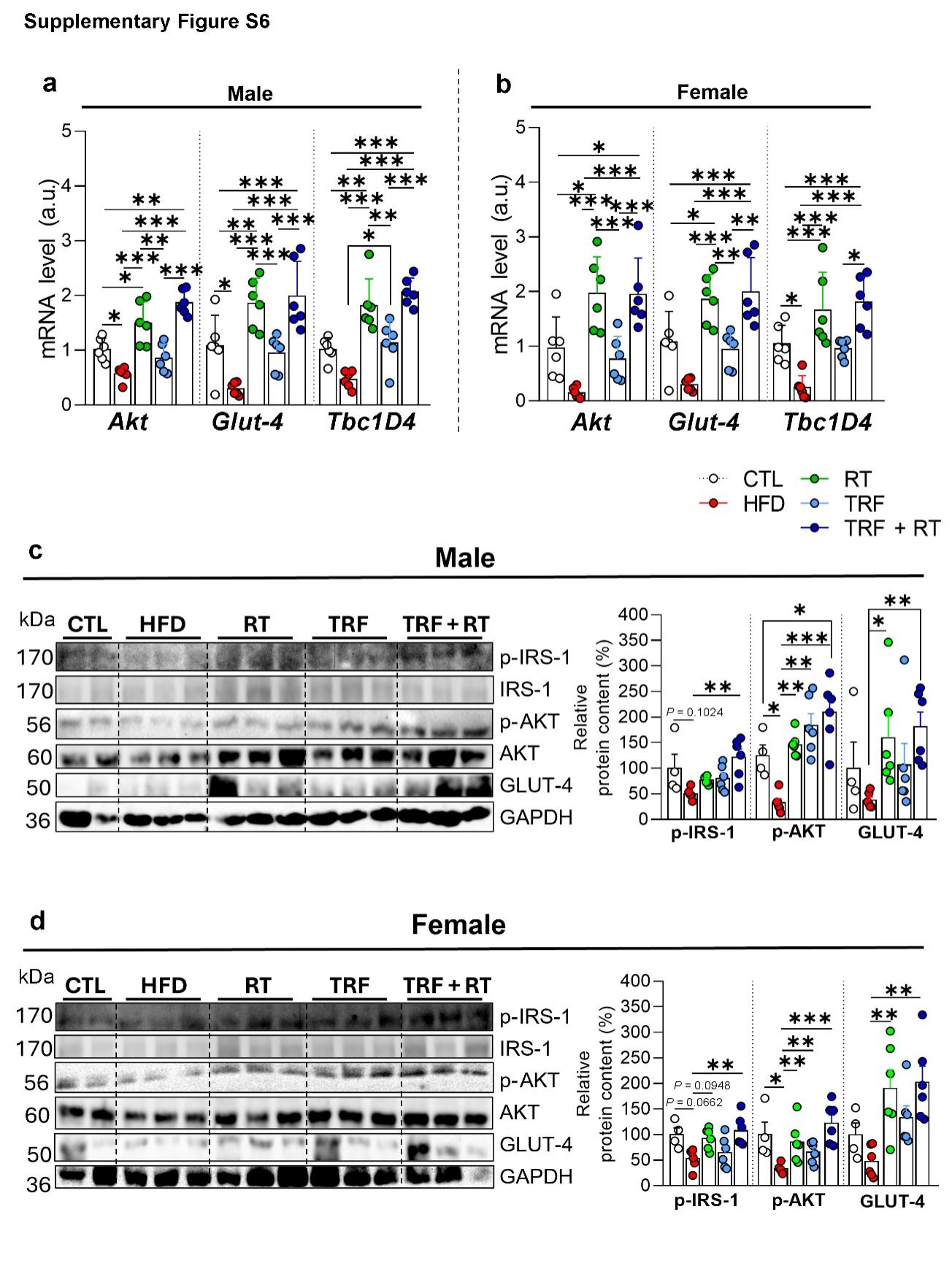


**Supplementary Figure S6** (a and b) mRNA expression related to insulin signaling pathway in the gastrocnemius of male and female mice. (c and d) Relative protein levels of p-IRS1^Tyr612^, p-Akt^Ser473^, and GLUT-4 total in the gastrocnemius muscle of male and female mice. Total IRS-1 protein and total Akt were used as assay controls for p-IRS-1 and p-Akt, respectively. The GAPDH protein was used as an assay control for total GLUT-4.For mRNA analyses (*n* = 5−6); for protein analyses: CTL (*n* = 4), HFD (*n* = 6), RT (*n* = 6), TRF (*n* = 6), and TRF + RT (*n* = 6). Statistical significance: ^*^*P* < 0.05; ^**^*P* < 0.01; ^***^*P* < 0.001.
